# Ancient Egyptian scribes and specific skeletal occupational risk markers (Abusir, Old Kingdom)

**DOI:** 10.1038/s41598-024-63549-z

**Published:** 2024-06-27

**Authors:** Petra Brukner Havelková, Veronika Dulíková, Šárka Bejdová, Jana Vacková, Petr Velemínský, Miroslav Bárta

**Affiliations:** 1 Department of Anthropology, National Museum in Prague, Cirkusová 1740, 193 00 Prague 9, Czech Republic; 2 Czech Institute of Egyptology, Faculty of Arts, Charles University, 110 00 Prague, Czech Republic; 3 Department of Anthropology and Human Genetics, Faculty of Science, Charles University, 128 00 Prague, Czech Republic; 4 Department of Applied Mathematics, Faculty of Information Technology, Czech Technical University in Prague, 160 00 Prague, Czech Republic

**Keywords:** Archaeology, Biological anthropology, Skeleton

## Abstract

Men with writing proficiency enjoyed a privileged position in ancient Egyptian society in the third millennium BC. Research focusing on these officials of elevated social status (“scribes”) usually concentrates on their titles, scribal statues, iconography, etc., but the individuals themselves, and their skeletal remains, have been neglected. The aim of this study is to reveal whether repetitive tasks and maintained postures related to scribal activity can manifest in skeletal changes and identify possible occupational risk factors. A total of 1767 items including entheseal changes, non-metric traits, and degenerative changes were recorded from the human remains of 69 adult males of well-defined social status categories from the necropolis at Abusir (2700–2180 BC). Statistically significant differences between the scribes and the reference group attested a higher incidence of changes in scribes and manifested themselves especially in the occurrence of osteoarthritis of the joints. Our research reveals that remaining in a cross-legged sitting or kneeling position for extended periods, and the repetitive tasks related to writing and the adjusting of the rush pens during scribal activity, caused the extreme overloading of the jaw, neck and shoulder regions.

## Introduction

The term “scribes” in relation to ancient Egypt in the third millennium BC covers men who held a wide range of administrative posts or functions. These individuals enjoyed a privileged position in society at that time, since only 1% of the population would have been literate^1^. A whole range of scribal titles associated with various departments or specific tasks is attested in the Old Kingdom^2^, but literacy and writing skills/scribal ability were an integral part of the employment of all administrative officials. Depending upon their hierarchical grade within a given office, it is supposed that the working activity of such officials in large part encompassed activities related to the writing of administrative documents. Those who headed a department (overseers) also managed its relevant agenda.

In recent years, scholars have directed their research towards various aspects associated with scribes, e.g., with their titles, scribal statues, iconography in wall decoration, writing materials, writing systems and analyses of single documents^2,3^. However, thorough research into the skeletal remains of these ancient Egyptian scribes, focusing on scribal activities and their possible influence on the development of activity-related skeletal changes, has been missing or limited to single case studies^4^.

Various skeletal markers manifesting on the human skeleton are supposed to be activity-induced in nature, and reflect a mechanical loading and repetitive tasks throughout life; and vice versa specific occupational physical activities may represent risk factors for the development of various changes to the skeleton. These include in particular the presence and character of entheseal changes (EC) at the insertion sites of tendons and ligaments^5,6^ or the size of muscle attachment sites^7^; selected non-metric traits (NMT) as accessory facets, notches, etc., on the infra-cranial skeleton^8–12^; degenerative changes, specifically osteoarthritis (OA) of joints of appendicular skeleton and vertebral column and intervertebral disc disease (IDD) at vertebral bodies^13–17^; and analysis of the cross-sectional geometric properties^18,19^.

In addition to the presumed effects of physical stress on the development of changes, more factors such as age, sex, metabolic disorders, or hormonal and genetic influences contribute to their aetiology. The link between physical activity and skeletal changes is still not entirely clear and is widely discussed^16,20–23^ also applies to osteoarthritic changes^16,24^. A clear association between degenerative changes and activity thus cannot easily be demonstrated^25,26^. Although the multifactorial aetiology of the aforementioned skeletal markers complicates the interpretations of the results, it seems that physical stress still remains among important factors influencing the development and character of change, but non-mechanical factors should also be controlled or discussed^20,22,23^.

**Figure 1:**
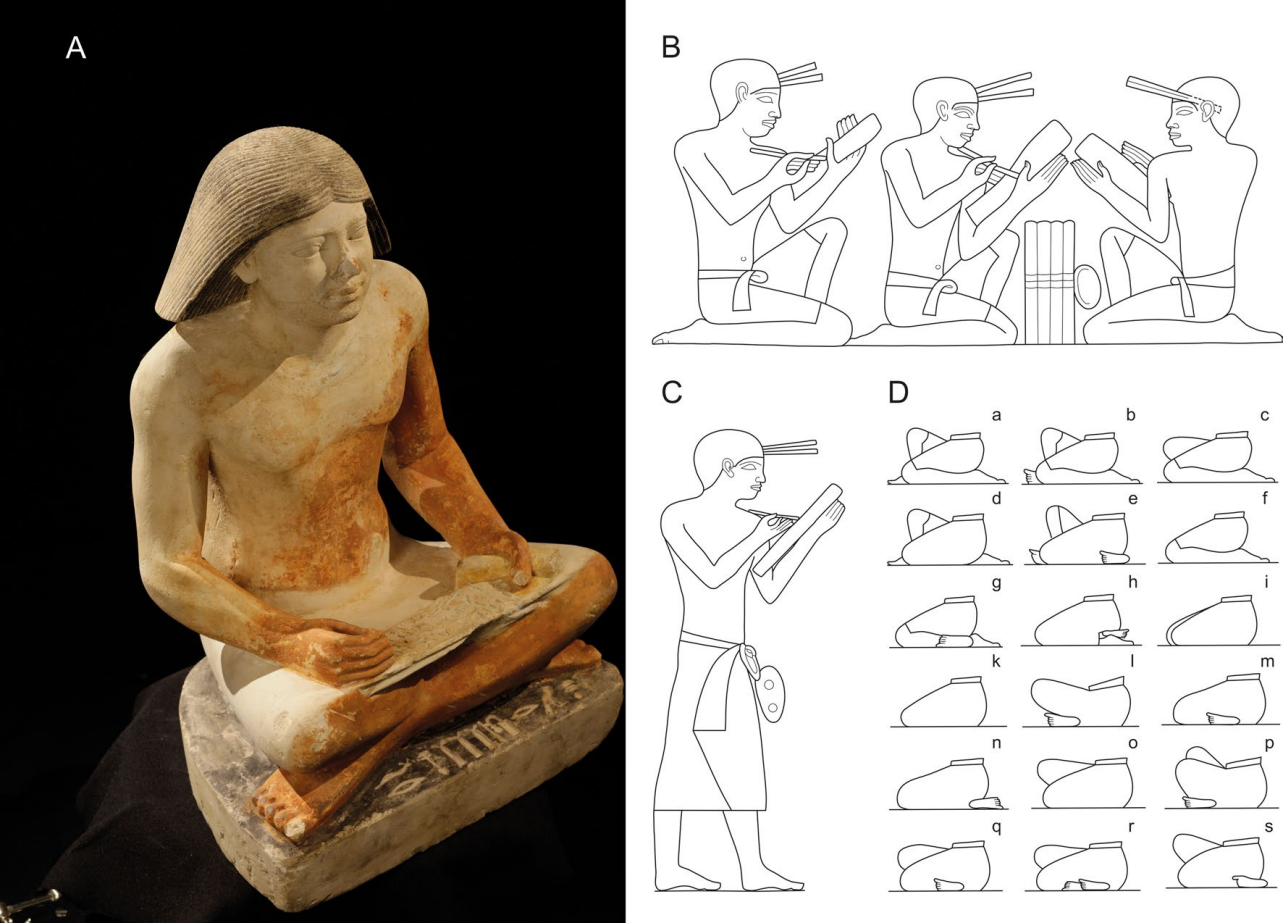
Working positions of scribes. (**A**) cross-legged (sartorial) position (the scribal statue of the high-ranking dignitary Nefer, Abusir; photo Martin Frouz); (**B**) kneeling-squatting position (wall decoration from the mastaba of the dwarf Seneb^29^); (**C**) standing position (wall decoration from the mastaba of the dwarf Seneb^29^); (**D**) based on tomb relief decoration, different position of the legs when sitting^30^. Drawing Jolana Malátková.

When attempting to reconstruct a possible pattern of habitual activities in individuals from past populations, the archaeological and/or historical context is crucial. For the scribes in ancient Egypt, there is evidence (textual, wall relief decoration in tombs and statues) that provides insights into the way working activities were carried out. Ancient Egyptian officials used a thin brush-like pen made of rush during the Pharaonic era, until it was substituted by the Greek-origin reed pen around 100 BC. They regularly wrote on papyrus, ostraca or wooden boards^27,28^. We also have a fairly accurate knowledge of the postures in which they performed their profession, and in which they spent relatively long periods. Simplistically, it may be assumed that they mainly adopted three typical poses: (a) the cross-legged position (sartorial), where the stretched skirt served as a table (Fig. 1A); (b) the kneeling-squatting position, one leg squatting with the second knee on the floor (usually seen in two-dimensional depictions; Fig. 1B ^29^); (c) the standing position (Fig. 1C ^29^). It is very likely that while specific positions varied (Fig. 1D ^30^), there could have been a general tendency for an individual to revert to a preferred position^31^. It may also have depended on the circumstances and the environment in which the scribal activity was carried out. Although, these positions and movements were by no means physically demanding, it can be assumed that daily repetition and maintaining them could affect specific areas of the skeleton.

In this paper we seek to reveal whether the repetitive tasks and positions related to scribal activity in Old Kingdom Egypt can manifest themselves in recorded changes in the skeleton, as well as which areas of the skeleton, i.e. attachment sites or joint articular surfaces, are significantly affected in comparison to the reference group. The identification of these areas, and especially their combinations, could then be useful in the identification of the “scribal profession” in the skeletons of those individuals whose titles have not been preserved—a practical outcome and the main aim of this and future research. We would also like to focus on identifying the most likely preferred position for scribes to perform their duties, and compare it with the archaeological evidence. To date, no analogous study has been published that comprehensively evaluates the occupational risk factors on the entire skeleton, while at the same time being based on a homogenous and relatively large sample of individuals whose employment was related to scribal activity.

## Results

Of the 1767 evaluated items in total, a statistically significant difference at the level of significance alpha 0.05 between the scribes and the reference group based on Mann–Whitney *U* test and/or Chi-square test was found in only 68 (3.85%); 34 significant differences were recorded on the skull and appendicular skeleton and 34 on the vertebrae. The results of the statistical analyses for these 68 traits are shown in Tables 1 and 2, separately for the skull and appendicular skeleton, and vertebrae (the complete results for all 1767 items are given in Supplementary Data S1 and Data S2). After adjustment using the Holm-Bonferroni correction, the number of statistically significant traits was reduced to 40. The traits that appeared non-significant after this correction were mainly scored on the lower limb and vertebrae. Although we recognize that the explanatory value of the differences between the scribes and the reference group is lower for those traits that were found to be significant using only the Mann–Whitney or chi-squared tests, we address them in the results and discussion.

**Table 1:** Overview and results of the statistical analysis of the evaluated traits on the skull and appendicular skeleton, where statistically significant differences between scribes and the reference group were revealed based on Mann–Whitney *U* test (M-W) and/or Chi-square test (Chi2). Confidence intervals (CI) at the 95% and 97.5% levels were calculated for odds ratios. The results of the Mann–Whitney U test for equality of age distributions between the scribes and the reference group (age M-W) are presented in the last two columns.

Bone	Location	Changes	Specification	Side	Scribes				Reference				M-W		Chi2		Chi2 odds ratio			Holm-Bonferroni	Age M-W	
					N	no	yes	%	N	no	yes	%	U	p	Stat	p	OR	CI 95%	CI 97.5%		U	p
Skull	Mandibular fossa	OA	SC	L	24	8	16	66.67	30	19	11	36.67	252	**0.031**	4.800	**0.028**	3.455	**1.12 to 10.67**	0.95 to 12.54	**TRUE**	260	0.062
		OA	NB	L	24	19	5	20.83	30	30	0	0.00	285	**0.010**	4.631	**0.031**	17.205	0.9 to 328.84	0.59 to 501.22	**TRUE**	260	0.062
		OA	OA	L	24	11	13	54.17	30	23	7	23.33	249	**0.021**	5.436	**0.020**	3.883	**1.21 to 12.47**	**1.02 to 14.74**	**TRUE**	260	0.062
	Condylar process	OA	MC	L	21	6	15	71.43	25	16	9	36.00	169.5	**0.018**	5.741	**0.017**	4.444	**1.27 to 15.52**	**1.06 to 18.57**	**TRUE**	190.5	0.090
		OA	SC	L	22	8	14	63.64	25	18	7	28.00	177	**0.016**	6.012	**0.014**	4.500	**1.31 to 15.42**	**1.1 to 18.4**	**TRUE**	194.5	0.066
		OA	JC	L	22	14	8	36.36	25	24	1	4.00	186	**0.006**	5.964	**0.015**	13.714	**1.55 to 121.42**	**1.13 to 166.08**	**TRUE**	194.5	0.066
		OA	OA	L	22	9	13	59.09	25	21	4	16.00	156.5	**0.003**	9.412	**0.002**	7.583	**1.93 to 29.72**	**1.59 to 36.16**	**TRUE**	194.5	0.066
		OA	MC	R	21	8	13	61.90	19	14	5	26.32	128.5	**0.027**	5.105	**0.024**	4.550	**1.18 to 17.52**	0.97 to 21.27	**TRUE**	124.5	0.**029**
		OA	OA	R	22	11	11	50.00	20	17	3	15.00	143	**0.018**	5.775	**0.016**	5.667	**1.28 to 25.02**	**1.04 to 30.96**	**TRUE**	149.5	0.056
Clavicle	Sternal facet	OA	SC	L	14	1	13	92.86	21	8	13	61.90	101.5	**0.045**	2.748	0.097	8.000	0.87 to 73.4	0.63 to 100.9	FALSE	83	0.**019**
		OA	MC	R	10	2	8	80.00	22	16	6	27.27	52	**0.007**	5.772	**0.016**	10.667	**1.74 to 65.27**	**1.34 to 84.66**	**TRUE**	64	0.044
	Acromial facet	OA	NB	R	9	4	5	55.56	17	16	1	5.88	38.5	**0.006**	5.621	**0.018**	20.000	**1.8 to 222.78**	**1.27 to 314.91**	**TRUE**	44	0.059
		OA	OA	R	9	1	8	88.89	17	11	6	35.29	35.5	**0.012**	4.816	**0.028**	14.667	**1.46 to 146.96**	**1.05 to 204.61**	**TRUE**	44	0.059
Scapula	Glenoid fossa	OA	SC	R	24	14	10	41.67	31	28	3	9.68	253	**0.006**	7.669	**0.006**	6.667	**1.58 to 28.16**	**1.28 to 34.64**	**TRUE**	256	0.**034**
Humerus	Lesser tubercle	EC	HSC (out)	L	10	5	5	50.00	21	18	3	14.29	64.5	**0.027**	2.840	0.092	6.000	**1.05 to 34.21**	0.82 to 43.93	FALSE	51	0.**015**
	Greater tubercle	EC	HSI (out)	R	10	6	4	40.00	15	15	0	0.00	45	**0.010**	4.477	**0.034**	21.462	**1.0 to 458.63**	0.65 to 710.29	**TRUE**	20	0.**001**
	Humeral head	OA	SC	R	21	13	8	38.10	28	27	1	3.57	192.5	**0.002**	7.376	**0.007**	16.615	**1.88 to 147.22**	**1.37 to 201.38**	**TRUE**	174.5	0.**010**
		OA	JC	R	21	16	5	23.81	27	27	0	0.00	216	**0.009**	4.851	**0.028**	18.333	0.95 to 353.35	0.62 to 539.23	**TRUE**	160	0.**006**
		OA	OA	R	20	12	8	40.00	28	25	3	10.71	198	**0.019**	4.128	**0.042**	5.556	**1.25 to 24.77**	**1.01 to 30.7**	**TRUE**	162	0.**008**
	Trochlea of humerus†	OA	MC	R	23	18	5	21.74	30	15	15	50.00	247.5	**0.038**	4.425	**0.035**	0.278	**0.08 to 0.94**	0.07 to 1.12	**TRUE**	187.5	0.**002**
Radius	Radial head	OA	SC	L	20	14	6	30.00	32	30	2	6.25	244	**0.023**	3.665	0.056	6.429	**1.15 to 35.95**	0.9 to 46.03	**TRUE**	206	0.**021**
	Lunate surface	OA	MC	L	19	5	14	73.68	26	17	9	34.62	150.5	**0.011**	6.706	**0.010**	5.289	**1.44 to 19.45**	**1.19 to 23.45**	**TRUE**	126.5	0.**003**
	Scaphoid surface	OA	MC	L	18	4	14	77.78	25	17	8	32.00	122	**0.004**	8.777	**0.003**	7.438	**1.85 to 29.96**	**1.51 to 36.59**	**TRUE**	123	0.**007**
Metacarpus	1st (proximal)	OA	SC	R	19	16	3	15.79	30	30	0	0.00	240	**0.028**	2.673	0.102	12.939	0.63 to 265.99	0.41 to 409.66	FALSE	201.5	0.065
		OA	OA	R	19	14	5	26.32	30	30	0	0.00	210	**0.004**	6.154	**0.013**	23.138	**1.2 to 447.34**	0.78 to 682.97	**TRUE**	201.5	0.065
	2nd (proximal)	OA	SC	L	19	16	3	15.79	27	27	0	0.00	216	**0.037**	2.338	0.126	11.667	0.57 to 240.37	0.37 to 370.33	FALSE	191.5	0.117
Pelvis	Ischial tuberosisty	EC	common origin	L	22	5	17	77.27	32	16	16	50.00	273	0.111	4.080	**0.043**	3.400	**1.01 to 11.45**	0.85 to 13.63	FALSE	260	0.085
	Acetabulum†	NMT	rounded edge pit	R	16	16	0	0.00	30	22	8	26.67	176	**0.026**	3.476	0.062	0.080	0.0 to 1.49	0.0 to 2.26	FALSE	195.5	0.275
Femur	Gluteal tuberosity†	EC	gluteus maximus	R	21	2	19	90.48	29	0	29	100.00	247	**0.031**	0.931	0.335	0.132	0.01 to 2.9	0.0 to 4.52	FALSE	190	0.**016**
	Medial condyle	OA	OA	R	21	14	7	33.33	30	27	3	10.00	241.5	**0.042**	2.915	0.088	4.500	**1.01 to 20.14**	0.81 to 24.98	FALSE	200.5	0.**019**
Patella	Superolateral part	NMT	vastus fossa	L	19	15	4	21.05	25	25	0	0.00	187.5	**0.019**	3.522	0.061	14.806	0.75 to 294.2	0.49 to 450.93	**TRUE**	156.5	0.**039**
		NMT	vastus fossa	R	20	13	7	35.00	23	23	0	0.00	149.5	**0.002**	7.219	**0.007**	26.111	**1.38 to 493.9**	0.91 to 751.7	**TRUE**	136.5	0.**014**
	Lateral surface†	OA	MC	R	14	12	2	14.29	23	12	11	47.83	107	**0.043**	2.950	0.086	0.182	0.03 to 1.0	0.03 to 1.28	FALSE	118.5	0.158
Talus	Upper part	NMT	medial squatting f	R	13	11	2	15.38	25	25	0	0.00	137.5	**0.047**	1.561	0.212	11.087	0.49 to 249.88	0.32 to 389.95	FALSE	117.5	0.145

*EC* entheseal changes, *NMT* nonmetric traits, *OA* osteoarthritis, *MC* marginal changes, *SC* surface changes, *NB* new bone, *JC *joint contour; *HSC (out)* outer part of m. subscapularis; *HIS (out)* outer part of m. supra-/infraspinatus *p* p-value; *U* M-W test statistic; † higher occurrence in the reference group. Statistically significant results for individual tests (p ≤ 0.05) are marked in bold. Statistically significant results after Holm-Bonferroni correction applied on the joint hypothesis (with the family-wise error rate ≤ 0.05) are marked as bold as TRUE.

Significants values are in bold.

**Table 2:** Overview and results of the statistical analysis of the evaluated traits on the vertebrae where statistically significant differences between scribes and the reference group were revealed based on Mann–Whitney (M-W) and/or Chi-square test (Chi2). Confidence intervals (CI) at the 95% and 97.5% levels were calculated for odds ratios. The results of the Mann–Whitney U test for equality of age distributions between the scribes and the reference group (age M-W) are presented in the last two columns.

Bone	Location	Changes	Specification	Side	Scribes				Reference				M-W		Chi2		Chi2 odds ratio			Holm-Bonferroni	Age M-W	
					N	no	yes	%	N	no	yes	%	U	p-value	stat	p	OR	CI 95%	CI 97.5%		U	p
Vertebral apophyseal joints (C1—S1 sup)																						
C1	Fovea	OA	SAG		20	3	17	85.00	31	12	19	61.29	213	**0.038**	3.292	0.070	3.579	0.86 to 14.87	0.7 to 18.25	FALSE	246.500	0.192
	Inferior	OA	MC	R	20	14	6	30.00	29	27	2	6.90	223	**0.035**	3.088	0.079	5.786	**1.03 to 32.49**	0.8 to 41.63	FALSE	223.000	0.147
C2	Dens	OA	OA		20	13	7	35.00	29	26	3	10.34	218.5	**0.039**	3.042	0.081	4.667	**1.03 to 21.07**	0.83 to 26.16	FALSE	214.500	0.102
	Inferior	OA	MC	R	15	8	7	46.67	24	21	3	12.50	118.5	**0.020**	4.002	**0.045**	6.125	**1.26 to 29.7**	**1.01 to 37.26**	**TRUE**	129.000	0.119
		OA	SC	R	15	11	4	26.67	24	24	0	0.00	132	**0.009**	4.529	**0.033**	19.174	0.95 to 386.9	0.62 to 594.31	**TRUE**	129.000	0.119
		OA	OA	R	15	11	4	26.67	25	25	0	0.00	137.5	**0.008**	4.741	**0.029**	19.957	0.99 to 402.28	0.64 to 617.85	**TRUE**	133.500	0.110
C3	Inferior	OA	MC	L	10	5	5	50.00	22	19	3	13.64	70	**0.032**	3.103	0.078	6.333	**1.11 to 36.0**	0.87 to 46.2	FALSE	66.000	0.060
		OA	SC	L	10	5	5	50.00	22	19	3	13.64	70	**0.032**	3.103	0.078	6.333	**1.11 to 36.0**	0.87 to 46.2	FALSE	66.000	0.060
		OA	Eb	L	10	8	2	20.00	22	22	0	0.00	88	**0.037**	1.901	0.168	13.235	0.57 to 304.96	0.37 to 477.4	FALSE	66.000	0.060
		OA	SAG	L	10	5	5	50.00	22	19	3	13.64	68.5	**0.028**	3.103	0.078	6.333	**1.11 to 36.0**	0.87 to 46.2	FALSE	66.000	0.060
C6	Superior	OA	MC	L	16	10	6	37.50	25	24	1	4.00	133	**0.006**	5.548	**0.019**	14.400	**1.53 to 135.51**	**1.11 to 186.98**	**TRUE**	120.500	**0.023**
C7	Superior	OA	MC	R	16	10	6	37.50	23	21	2	8.70	131	**0.032**	3.197	0.074	6.300	**1.07 to 36.94**	0.83 to 47.61	FALSE	101.500	**0.012**
	Inferior	OA	OA	R	16	9	7	43.75	23	20	3	13.04	127.5	**0.035**	3.195	0.074	5.185	**1.08 to 24.79**	0.87 to 31.04	FALSE	101.500	**0.012**
T1	Inferior	OA	MC	R	14	3	11	78.57	23	17	6	26.09	76.5	**0.002**	9.653	**0.002**	10.389	**2.14 to 50.43**	**1.71 to 63.27**	**TRUE**	113.000	0.111
T2	Superior†	OA	SC	L	18	18	0	0.00	21	15	6	28.57	135	**0.016**	4.081	**0.043**	0.064	0.0 to 1.24	0.0 to 1.89	**TRUE**	113.000	**0.021**
T7	Superior	OA	SC	L	15	11	4	26.67	20	20	0	0.00	110	**0.017**	3.675	0.055	16.043	0.79 to 325.39	0.51 to 500.18	**TRUE**	91.500	**0.039**
		OA	MC	R	16	11	5	31.25	20	19	1	5.00	118	**0.041**	2.723	0.099	8.636	0.89 to 83.75	0.64 to 116.05	FALSE	100.500	**0.045**
		OA	SC	R	16	11	5	31.25	20	19	1	5.00	118	**0.041**	2.723	0.099	8.636	0.89 to 83.75	0.64 to 116.05	FALSE	100.500	**0.045**
		OA	OA	R	16	13	3	18.75	20	20	0	0.00	130	**0.050**	2.005	0.157	10.630	0.51 to 222.62	0.33 to 343.79	FALSE	100.500	**0.045**
T8	Inferior	OA	MC	L	15	8	7	46.67	22	20	2	9.09	103	**0.010**	4.952	**0.026**	8.750	**1.49 to 51.5**	**1.15 to 66.43**	**TRUE**	107.000	0.057
L1	Inferior	OA	MC	L	18	9	9	50.00	21	18	3	14.29	121.5	**0.018**	5.804	**0.016**	6.000	**1.3 to 27.77**	**1.04 to 34.6**	**TRUE**	113.500	**0.023**
		OA	MC	R	15	8	7	46.67	20	18	2	10.00	95	**0.016**	4.266	**0.039**	7.875	**1.33 to 46.63**	**1.03 to 60.19**	**TRUE**	66.000	**0.003**
L2	Superior	OA	MC	L	18	10	8	44.44	17	15	2	11.76	103	**0.037**	3.114	0.078	6.000	**1.05 to 34.32**	0.82 to 44.08	FALSE	91.000	**0.029**
L4	Superior	OA	MC	L	17	3	14	82.35	21	13	8	38.10	99.5	**0.007**	7.549	**0.006**	7.583	**1.65 to 34.9**	**1.32 to 43.46**	**TRUE**	107.500	**0.027**
	Inferior	OA	MC	R	13	4	9	69.23	19	16	3	15.79	57.5	**0.003**	7.264	**0.007**	12.000	**2.18 to 66.03**	**1.71 to 84.35**	**TRUE**	77.000	0.061
L5	Superior	OA	MC	R	13	2	11	84.62	20	11	9	45.00	78.5	**0.026**	5.179	**0.023**	6.722	**1.17 to 38.5**	0.91 to 49.47	**TRUE**	75.000	**0.031**
		OA	OA	R	14	6	8	57.14	20	16	4	20.00	88	**0.030**	3.481	0.062	5.333	**1.16 to 24.47**	0.93 to 30.45	FALSE	84.500	**0.039**
	Inferior	OA	MC	L	14	7	7	50.00	18	15	3	16.67	84	**0.050**	2.669	0.102	5.000	0.99 to 25.34	0.78 to 31.99	FALSE	72.000	**0.030**
Vertebral bodies (C2 inf—S sup)																						
C3	Inferior	IDD	SP		14	4	10	71.43	25	17	8	32.00	106	**0.020**	5.614	**0.018**	5.313	**1.27 to 22.24**	**1.03 to 27.32**	**TRUE**	100.500	**0.020**
		IDD			14	5	9	64.29	27	19	8	29.63	123.5	**0.036**	4.562	**0.033**	4.275	**1.09 to 16.83**	0.89 to 20.49	**TRUE**	104.000	**0.013**
T3	Superior	IDD	SP		11	6	5	45.45	19	17	2	10.53	68	**0.035**	2.999	0.083	7.083	**1.07 to 46.68**	0.82 to 61.19	FALSE	88.500	0.470
T4	Inferior†	EC			13	12	1	7.69	19	8	11	57.89	61	**0.005**	6.296	**0.012**	0.061	**0.01 to 0.57**	**0.0 to 0.78**	**TRUE**	72.000	**0.036**
L2	Superior†	IDD	SC		18	18	0	0.00	21	16	5	23.81	144	**0.030**	3.016	0.082	0.081	0.0 to 1.58	0.0 to 2.42	FALSE	121.500	**0.042**
L4	Superior	EC			16	8	8	50.00	21	17	4	19.05	116	0.052	3.970	**0.046**	4.250	0.98 to 18.4	0.8 to 22.7	FALSE	98.000	**0.023**

*EC* entheseal changes, *OA* osteoarthritis, *MC* marginal changes, *SC* surface changes, *Eb* eburnation, *SAG* Sager’s method, *IDD* intervertebral disk disease, *SP* spondylosis, *p* p-value,*U* M-W test statistic; † higher occurrence in the reference group. Statistically significant results for individual tests (p ≤ 0.05) are marked in bold. Statistically significant results after Holm-Bonferroni correction applied on the joint hypothesis (with the family-wise error rate ≤ 0.05) are marked as bold as TRUE.

Significants values are in bold.

The observed statistically significant differences indicate a higher incidence of changes in scribes compared to the reference sample (61/68; 90%; respective 37/40; 92.5% after Holm-Bonferroni correction) and manifested themselves especially in the occurrence of degenerative changes to the joints (OA and/or minor criteria) for both the appendicular skeleton and vertebrae.

**Figure 2:**
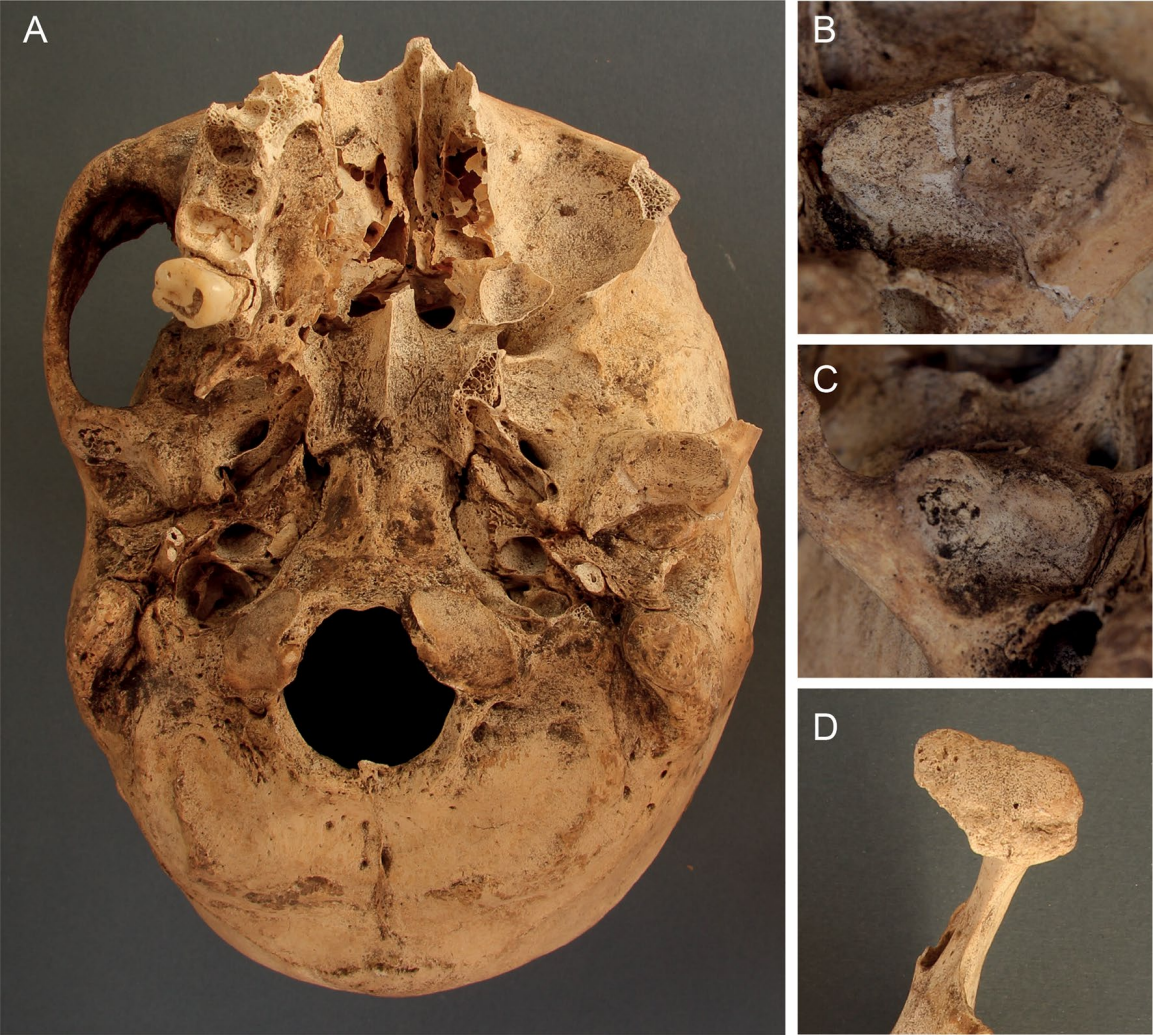
Osteoarthritis of the temporomandibular joint of a supposed family member (174/AS79/2015) of Khemetnu, the presumed owner of family tomb AS 79. Khemetnu’s working activity was associated with the household management and the arranging of the funerary cult of the judge Inti (AS 22). (**A**) Base of the skull with both mandibular fossae exhibiting OA; (**B**) Eburnation in the left mandibular fossa; (**C**) Subchondral lesions in the right mandibular fossa; (**D**) Joint contour deformation and marginal changes of the left condylar process. Photo Šárka Bejdová.

**Figure 3:**
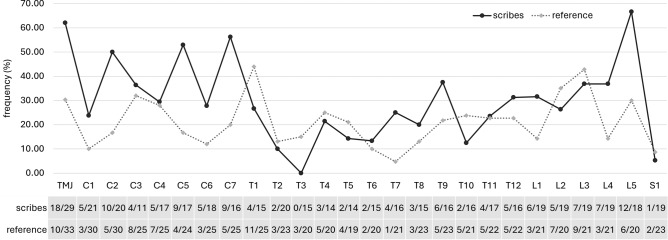
Comparison of the incidence of osteoarthritis (OA) in the temporomandibular joint (TMJ) and the apophyseal joints of the vertebrae (C1-S1) between scribes and the reference group (OA for the whole joint/vertebra was scored as present if OA occurred in at least one articular surface of an apophyseal joint). The number of individuals with OA (presence of OA/total number of individuals evaluated) is shown in the table below the graph.

**Figure 4:**
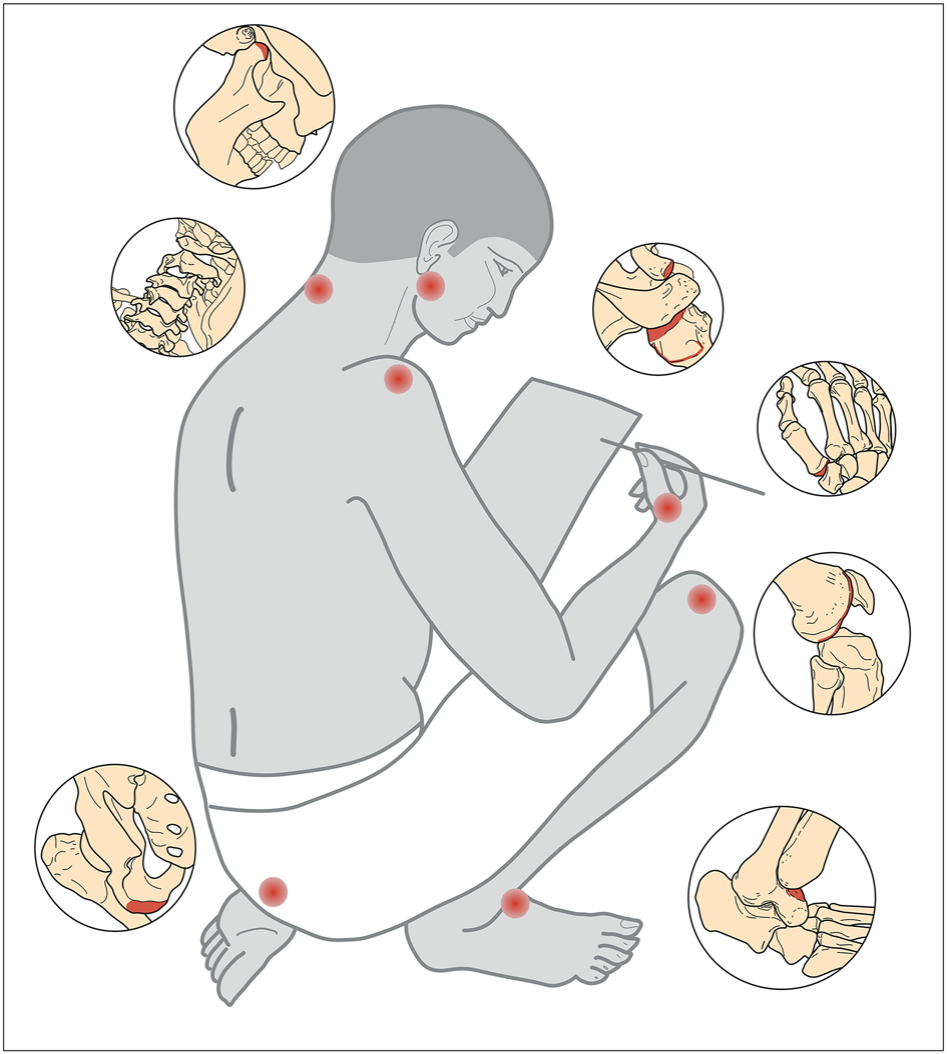
Drawing indicating the most affected regions of the skeletons of scribes with higher prevalence of evaluated changes compared to reference group: both temporomandibular joints (OA); cervical spine (OA, spondylosis); right shoulder (OA of the acromial facet of the clavicle and humeral head, EC on the greater tubercle of the humerus); right first metacarpal bone (OA); left ischial tuberosity (EC); right femoral medial condyle (OA) and the medial squatting facet on the right talus. Drawing Jolana Malátková.

In terms of the OA changes on the skull and appendicular skeleton in scribes, a higher incidence was observed at the areas of both temporomandibular joints (Figs. 2, 3), the right clavicle, the right humeral caput, proximal articular surface of the right first metacarpal bone (CMC1) and the medial condyle of the right femur (Fig. 4). Differences in minor changes (SC, MC, JC, NB) were also recorded on other joints of the appendicular skeleton (see Table 1), almost exclusively more frequently in scribes. Only two minor osteoarthritic changes showed a higher incidence in the reference sample: marginal changes (MC) on the right lateral surface of the patella and the trochlea of the humerus.

As for the vertebral column, statistically significant differences of OA changes of apophyseal joints were more common than the alterations of vertebral bodies, i.e., intervertebral disc disease (IDD; Table 2). All significant OA changes including the minor criteria occurred more often in scribes, except for the left upper facet of the T2 vertebra. A higher prevalence of OA in scribes was observed especially on the cervical vertebrae (C1, C2, C3, C7) and the T7 and L5 vertebra (Fig. 3). Minor OA changes were also noted on other vertebrae (Table 2). Differences in the incidence of IDD were recorded only on the lower surface of the C3 vertebra and the upper surface of the T3 vertebra, with a higher prevalence in scribes, and surface changes of the L2 upper surface in the reference sample.

A statistically significantly higher incidence of EC in the scribes was noted at two attachment sites on the humerus (the margin of the left entheses of the subscapularis; HSC Out, and the right infra-/supraspinatus; HSI Out) and on the left hip bone (ischial tuberosity), while in the reference sample only on the right femur (gluteus maximus). In the case of vertebral bodies, EC on the inferior part of the T4 body were significantly higher in the reference sample, while on the superior part of the L4 in scribes.

Regarding NMT, vastus fossa on the both patellae and medial squatting facet on the right talus, were recorded more often in scribes, while a rounded edge pit on the right acetabulum was more typical for the reference sample.

The results of the statistical tests did not indicate significant differences in the age-at-death distribution between all the scribes and the reference group when all individuals evaluated were included (χ^2^ = 0.208 < χ^2^α=0.05 = 5.991; Mann–Whitney: Z = − 0.242, p-level = 0.809). However, due to differences in the preservation of the traits assessed, the representation of scribes and reference individuals in each age category varies for each trait. The consistency of the distribution of individuals in the age categories was therefore monitored separately for each trait using the Mann–Whitney *U* test (see last two columns of the Tables 1 and 2 for selected traits; Supplementary Data S1 and Data S2 for complete results). These results reveal a different age distribution between scribes and the reference group for 35% of the traits evaluated (Supplementary Data S1 and Data S2). Traits for which we found a statistically significant difference between scribes and the reference group show a different age distribution in half of the cases (see Table 1 and Table 2). Typically, there is a higher number of older individuals among the scribes. The possible influence of age on the observed differences should thus be taken into account for these traits.

All evaluated markers were tested for side asymmetry by Wilcoxon Matched Pairs Test, separately for scribes and the reference sample. Statistically significant differences between the right and left sides were noted only for three traits in the scribes—EC: biceps brachii insertion on the radius (N = 12; T = 0.000; Z = 2.023; p-level = 0.043), OA: mandibular fossa on the mandible (N = 22; T = 0.000; Z = 2.023; p-level = 0.043), NB: trochlea of humerus (N = 20; T = 0.000; Z = 2.023; p-level = 0.043) and three traits in the reference group – EC: ischial tuberosity on the pelvis (N = 18; T = 0.000; Z = 2.201; p-level = 0.028); NMT: lateral extension of the trochlear surface on the talus (N = 24; T = 0.000; Z = 2.023; p-level = 0.043), SC: glenoid cavity on the scapula (N = 31; T = 0.000; Z = 2.201; p-level = 0.028).

## Discussion

Professions involving scribal agenda was certainly not physically demanding, but neither can it be assumed that individuals of lower status buried at Abusir performed physically demanding activities on a daily basis. These were not labourers, but usually members of the household or relatives, performing activities related to the running of the household^32,33^. The similar lifestyle of the two studied samples was manifested in the low number of evaluated traits showing statistically significant differences (3.85%), most of them showing a higher incidence among scribes (90%).

These statistically significant differences are in most cases reflected in the occurrence of degenerative joint changes. The intensity of these traits is usually attributed to increasing age^14,16,34,35^. Although the statistical tests did not reveal any significant differences in the age-at-death distribution between the compared groups, the possible influence of advanced age in the scribesʼ group on the higher incidence of degenerative changes cannot be completely ruled out, because for half of the traits for which statistically significant differences were found, the distribution across age categories is not the same—scribes tend to be older. Simplistically, we can conclude that these differences do not apply to the temporomandibular joint region, the acromial facet of the clavicle, the wrist and metacarpus, the pelvic and talar traits, and the upper cervical and mid-thoracic spine, where the distribution of individuals in the age categories is similar. Conversely, the observed differences in the prevalence of changes assessed in the humerus, femur, and patella, as well as the lower cervical and lumbar spine, may be influenced by this unequal age distribution. However, in the study presented it would be highly likely that statistically significant differences in the incidence of degenerative changes between scribes and the reference group would occur in a much larger number, or even the majority, of the articular surfaces assessed, rather than in a minority of them, localised to a few regions of the skeleton. This permits the assumption that the observed differences could to some extent be related to the scribal activity associated with well-defined repetitive tasks and stationary posture.

One of the areas significantly more affected in the group of scribes was the spine, especially in the **cervical region**. Two separate areas of vertebrae were examined: (zyg)apophyseal joints with osteoarthritic changes (OA/minor criteria) and intervertebral joints (bodies) with marginal spondylosis (SP) and surface changes (IDD). The prevalence of changes in a particular vertebra is different for each marker; they are basically uncorrelated, which is due to the different function of the apophyseal and intervertebral joints of the spine. The intervertebral joints allow a minimum of movement and provide support, while the apophyseal joints provide less support with varying degrees of movement.

Compared to previous studies^15,36–38^, the curves characterizing the degree of loading of individual vertebrae in the present study approximately correspond for both scribes and the reference sample, but some atypical manifestations are worth mentioning. All cervical vertebrae show more degenerative changes in the scribes compared to the reference group, but the statistically significant differences concern mainly OA at the apophyseal joints. The C7 vertebra exhibited the highest incidence of OA changes at apophyseal joints among the cervical vertebrae, and significantly exceeded the frequency in the reference sample and frequencies recorded in previous studies. The location of the C7 vertebra at a point where the lordotic curvature of the cervical spine ends and the thoracic kyphosis begins implies that the lower facets are under continuous tension, partially due to the action of the nuchal ligament that ends at the spinous process of the C7^37^. The C7 vertebra is thus usually one of the most affected vertebrae in OA^25^. This explains the frequent occurrence of OA at the C7 vertebra, but not the enormous increase in OA in scribes compared to the reference group. It cannot be ruled out that this could be related to the constant overloading of the cervical spine.

In a typical scribe’s working position, the head had to be forward, and the spine flexed, changing the centre of gravity of the head and putting stress on the spine. An exaggerated dorsal kyphosis (round back) places the head ahead of the centre of gravity, increasing the cervical lordosis. The weight of the head in this position is borne by the apophyseal joints^38^. The forward head posture with a flexed neck is a position characteristic of many of modern occupations. In a sitting work position characterized by flexion of the whole spine to accommodate the eye-object distance to a horizontal working surface (as with scribes), the load moment for the C7-T1 motion segment induced by the weight of the head and neck increases 3.6 times in comparison to the neutral position^39^. Prolonged cross-legged sitting could result in a significant increase of degenerative changes to the cervical spine among scribes.

Atypical and excessive loading of the cervical spine in the group of scribes is also accompanied by the extreme prevalence of OA in the **temporomandibular joint** (TMJ). The prevalence of TMJ OA ranges from 16% to 38% in living populations and from 2.4% to 40% in archaeological samples^40–42^. The prevalence of TMJ OA in individuals in the reference sample (30%) falls exactly within this range. However, the temporomandibular joints of the scribes were affected far beyond the reported incidence of TMJ OA in previous studies (64%). The development of TMJ OA is most commonly associated with dental pathologies, dental attrition or antemortem tooth loss of the posterior dentition^40^ or with dietary habits^43^. The significant loading of the TMJ could also be closely related directly to scribal activity. The tools that scribes used to write were made from rush (*Juncus*), which was cut at a slant and chewed at the end to form a brush-like head^28^. When the pen became ragged or clogged with ink, the scribe cut off the end again and chewed the next section. Frequent or long-term chewing may be a risk factor for TMJ disorders with a high percentages of osteoarthritis^44^. This scribal habit of retting by chewing the rush brush used to write hieroglyphs might also have led to the asymmetrical dental wear evidenced in the mandible of the scribe Horemkenesi (21st Dynasty, Thebes), where it resulted in mesio-distal depressions along the molar teeth^45^.

However, the existing relationship between the load on the cervical spine and the temporomandibular joint cannot be ignored. The correlation between temporomandibular disorders and cervical spine dysfunction or neck/shoulder area symptoms is well documented or reviewed by clinical studies^46–49^. The TMJ and cervical spine are anatomically and functionally interrelated, and the malposition of one can affect the position and function of the other^50^. Postural maintenance of the head is achieved through a muscle chain which is formed, among others, by the large posterior neck muscles and anterior muscle attachments to the mandible and hyoid bones, as they link the cranium to the shoulder girdle. Any alteration of this functional chain anywhere along its course will be reflected elsewhere along its length^48^. Therefore, we cannot exclude that the high incidence of TMJ OA among scribes compared to the reference sample may to some extent be related to extreme overloading of the cervical spine. While scribes probably alternated the position of the arms and leg, the head and cervical spine remained in a forward position, resulting in excessive strain. It should be noted that in the case of the temporomandibular joint and upper cervical vertebrae, no statistically significant differences in age distribution were found between the scribes and the reference group. Therefore, in this case, the differences observed between the groups of individuals evaluated are not due to differences in age distribution.

**Figure 5:**
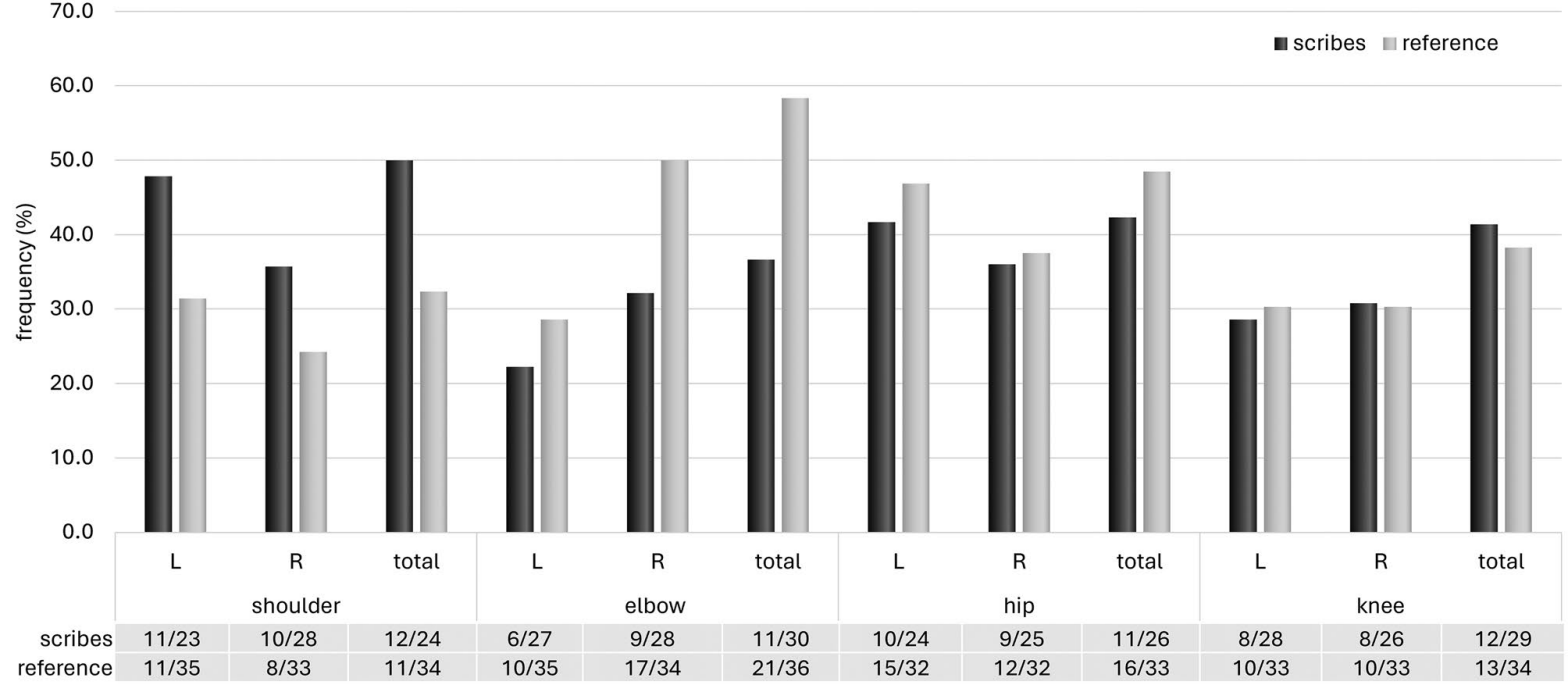
Comparison of the incidence of osteoarthritis (OA) in large joints (shoulder, elbow, hip, knee) between scribes and the reference group (OA for the whole joint was scored as present if OA.

As in the case of the cervical spine and TMJ, statistically significant differences in the shoulder and the whole **upper limb** were higher in the group of scribes for most of the studied traits. Nevertheless, statistically significant differences were observed for only a few traits, linked mainly to OA or its minor criteria. The scribes’ group manifested a significant increase in OA changes at the right shoulder (humeral head, acromial facet of clavicle) and the right first metacarpal bone. The pattern of right shoulder loading is complemented by an increased occurrence of entheseal changes at the margin of the supra- and infraspinatus muscle attachment on the humeral greater tubercle (HSI), which belongs to the rotator cuff group. The prevalence of shoulder osteoarthritis is usually low comparison to other large joints^34,51,52^. The shoulder and hip are usually less severely involved than the knee or elbow^15^. By contrast, among scribes the shoulder is the most affected joint compared to the hip and knee (unlike the reference group, where it was the elbow area; Fig. 5). However, apart from the acromial facet of the clavicle and the first metacarpal, the age distribution of the scribes differs from that of the reference group for the traits evaluated, with the scribes having a greater number of older individuals. The influence of age cannot be excluded in this case.

In conjunction with the involvement of the supra- and infraspinatus muscle tendons, this could indicate an overall overloading of the shoulder region. Stress to the rotator cuff, specifically the supraspinatus muscle, usually occurs when the arm is in an elevated position, such as among painters^53^. However, it could also be related to a static sitting position with arms unsupported, such as during typing^54,55^. Last but not least, the shoulder area is connected to the cervical spine, both functionally and by innervation. Especially in the C7/T1 joint area, where an enormous load was recorded among scribes, the upper girdle and vertebral column are linked^25^. Studies focused on the impact of disorders in the ‘upper quarter’ of the body on posture and pain experienced in different parts of the body have found a relationship between the presence of signs and symptoms in the jaw and neck/shoulder areas^46,49^.

Based on the iconography and statues, it can be assumed that scribes wrote with their right hands and rolled the papyrus scroll left-handed. A papyrus 6 m length, when tightly rolled, yielded a cylinder 5–6 cm thick, which could easily be spanned by the fingers of a hand^28^. Writing with a rush pen required dexterity that allowed the creation of clearly written ligatured as well as non-ligatured signs of l cm or less^28^. These repetitive movements and positions can lead to excessive strain on the wrists and hands, which could be reflected in changes to the bones. Occupational risk factors of the hand and wrist are however usually closely related to tendon-related disorders (tendinitis, carpal tunnel syndrome, etc.) caused by the chronic performance of forceful exertions or highly repetitive hand-intensive tasks^56^. These work-related musculoskeletal disorders are difficult to identify on the skeleton and to interpret appropriately. As for the wrist area of the studied skeletons, only minor osteoarthritic changes (MC, SC) exhibited a significantly higher incidence in the group of scribes, on both hands localized to the scaphoid and lunate area. Unlike tendon-related disorders, OA of the wrist is usually traceable to a secondary to posttraumatic sequel, such as fracture, dislocation, ligament injury or metabolic disease^57,58^. Although there are several studies referencing an association between wrist/hand OA and specific movements (pinch grip, power grip, impact loading), occupations or kinds of activity^35,59–61^, it is still very difficult to define how a scribal activity would affect such a complex joint as the wrist and hand.

In addition to the minor changes on the aforementioned articular surfaces, a significantly higher occurrence of OA on the proximal articular surface of the right first metacarpal bone (CMC1) was detected among scribes. This could be related to specific thumb motions, their frequency and duration, and specific thumb positions, which may constitute long-term low-level mechanical stresses, resulting in degenerative changes in the articular cartilage and initiating OA^62^. The development of OA of the CMC1 has been shown to be closely associated with pinch grip work especially^61,63^, which corresponds well to the repetitive use of the right hand during scribal activity. However, further research focusing on other musculoskeletal markers (e.g., entheseal changes) in the wrist and hand will be needed, following some of the current methods^64,65^.

One of the initial hypotheses was that differences among groups would manifest themselves in the **lower part of the body**, especially in the area of the knees due to the kneeling working position. However, this assumption was not confirmed. After adjusting the data by Holm-Bonferroni correction, the only significant trait was the vastus fossa on the patella, the presence of which is problematic in terms of interpretation due to unclear aetiology and the lack of literature. Its higher incidence in scribes may also be associated with heredity^66^, given the familial nature of the tombs.

Focusing only on the significant results of the Mann–Whitney test, individuals from both groups also exhibited similar degrees of OA changes at the knee and the hip region (Fig. 5). However, OA on the medial condyle of the right femur manifested significantly more among scribes. During knee flexion there is a load imbalance between medial and lateral tibiofemoral contact forces, created by an adduction moment in the knees^67^. Contact stresses increase significantly during deep knee flexion and medial peak pressures are greater than pressures in the lateral tibiofemoral compartment^68^. Occupational kneeling (e.g., for floor layers) also increases the risk of degenerative tears in the medial, but not the lateral, menisci^69^. Therefore, it cannot be excluded that the OA on the right medial condyle of the femur is related to repetitive deep knee flexion in the kneeling posture of scribes. Given the right-sided loading of the joint in question, it is possible that they preferred kneeling/squatting on the right knee, which would correspond with a higher incidence of the medial squatting facet on the right talus among scribes. The other probable preferred position, cross-legged sitting, could put more pressure on the buttock, especially the ischial tuberosity where the common origin of the biceps femoris, semitendinosus and semimembranosus is located. This enthesis on the left side manifested a significantly higher occurrence of ECs in the scribes’ group compared to the reference sample. The aforementioned muscles are mainly involved in thigh extension at the hip joint, in flexion of the leg at the knee joint, and rotation of the leg with flexed knee^70^, which corresponds to the cross-legged siting position. Bioarchaeological studies focusing on the relationship between the intensity of physical loading and the occurrence of EC on the ischial tuberosity rate this correlation as very weak and they cite age as a major factor in their occurrence^70,71^. However, in the present study there was no difference in age distribution for this trait between the scribes and the reference group. Clinical studies, particularly from Asia where cross-legged sitting position is still frequently used, have highlighted the health risks associated with this position when the lower legs are folded and crossed at the ankles or calves^72^. Gluteal/buttock pressure varies depending on the position of the lumbar spine (lordosis, flat, slump); anterior and posterior tilts of the upper body in the slump and lordosis postures may result in more pressure concentration in the feet or buttocks, respectively^72,73^. Increasing gluteal pressure could thus be one of the factors related to the changes found in the ischial tuberosity among scribes. Cross-legged sitting positions also significantly alter all of the spinopelvic alignment—the lumbar lordotic angle, sacral slope, pelvic tilt, and pelvic incidence^74^—which may lead to a reduction in lumbar lordosis (becoming more kyphotic) and an increased compression load, especially at the L4-5 vertebrae^72,75^. This may explain the higher minor OA changes on the lumbar vertebrae (and OA in L4) in the group of scribes, but the effect of greater age cannot be ruled out, as in the case of the medial condyle of the right femur.

Nevertheless, if we accept that the changes observed in the lower limbs of scribes (EC on the left ischial tuberosity, OA on the right femoral medial condyle, the medial squatting facet on the right talus) are related to their activity, then this would indicate a preferred position where the left leg is in a kneeling or cross-legged sitting position and the right leg in a squatting position as visible in Fig. 1B and D (a, d) and Fig. 4.

The overall non-significant differences between the scribes and the reference group in the lower limb region may also indicate that kneeling or a cross-legged sitting position was a common posture in the Old Kingdom, unrelated to any specific occupation, as reflected in various tomb scenes where family members and dependents are sitting on the floor and in so-called “servant statues” depicting people performing everyday tasks^33^.

The present research provides a first insight into possible occupational risk factors related to scribal activity and the most affected areas of the scribes’ skeletons. Further research will focus on the examination of selected skeletal regions where significant differences have been recorded and on evaluation of a similar population (e.g., Giza).

## Conclusions

- The scribes and reference group significantly differ in only 3.85% of all the evaluated skeletal traits, which might reflect an overall likeness (male sex, identical age distribution) and similarity in their lifestyle, including the absence of physically demanding activities in both groups.
- Most of these statistically significant differences, show a higher prevalence of the observed changes among the scribes (90%), which allows us to deduce that they might be related to scribal activity, but the significant effect of age cannot be ruled out for some traits, for which an unequal age distribution between scribes and the reference group was recorded.
- The different kinds of changes (OA, EC, NMT) recorded at a significantly higher frequency among scribes were clustered in a few well-defined regions or structures of the skeleton: the temporomandibular joint, the cervical spine, the right shoulder, the right first metacarpal bone, the femoral medial condyle of the right knee, the left ischial tuberosity and the right talus (Fig. 4).
- Upper body region: the occurrence of TMJ OA is probably related to the excessive and frequent chewing of rush pens by scribes, but in addition TMJ disorders are often related to extreme loading of the cervical spine and vice versa. The latter is also significantly loaded in the scribes’ group, probably in relation to prolonged forward head positioning in a sitting or kneeling posture during scribal activity. The loading of the shoulder area may also be connected to the cervical spine, but the observed changes could also be related to a static sitting position with arms unsupported, such as during scribing. The development of OA of the right first metacarpal bone probably reflects pinch grip work, such as frequent gripping of the pen.
- Lower body region: the higher incidence of changes in the aforementioned traits (right knee, left ischial tuberosity, right talus) among scribes compared to the reference group, and their combination, could indicate a preferential position for scribes where the left leg is in a kneeling or cross-legged sitting position and the right leg in a squatting position, which could also cause overloading of the lumbar spine.
- Risk factors for the occupation of scribe very likely did not include kneeling or squatting, which were probably also common in the rest of the population and could be alternated with standing, but extreme overloading of the neck and shoulder region.

## Materials and methods

### Sample of study

At its height in the age of the pyramid builders (the third millennium BC), kings and royal family members (5th Dynasty) and non-royal elite (5th and 6th Dynasties) built their tombs in the necropolis at Abusir where archaeological research is carried out by the Czech Institute of Egyptology (Faculty of Arts, Charles University, Prague) since 1960. To date, almost two hundred tombs dating back to the Old Kingdom (2700–2180 BC) have been discovered. Although Abusir is perceived primarily as a burial ground of the Old Kingdom elite population, the arrangement of tombs within the necropolis reflects differences in the social status of their owners^76^. Although the first skeletal remains of Old Kingdom individuals were unearthed in the Abusir region in 1976, the process of collecting sufficient material for a “population” study took several decades^77^. Research focusing on the health status and activity related markers on the scribes’ skeletons began in 2009, but it took another 10 years to uncover enough skeletons for a comprehensive study. Currently, the Abusir skeletal collection contains 221 Old Kingdom individuals, of which 102 are estimated to be male; 26 male skeletons from previous research have been reburied and therefore could not be evaluated for this study.

Unambiguous dating to the Old Kingdom period, precise social status determination and successful sex and age-at-death estimation were the basic criteria for the inclusion of an individual in the assemblage. Our study includes the human remains of 69 adult males of different age-at-death and social status categories (Supplementary Data S3). The complete numbers and frequencies of individuals according to age-at-death for both groups (scribes and reference group) are summarized in Table 3.

**Table 3:** Absolute numbers (N) and frequencies (%) of males in the scribe and reference groups according to age-at-death categories.

	^scribes^		^reference^		^total^	
	^N^	^%^	^N^	^%^	^N^	^%^
^YA [20–35]^	^6^	^20.0^	^9^	^23.1^	^15^	^21.7^
^MA [35–50]^	^11^	^36.7^	^15^	^38.5^	^26^	^37.7^
^OA [50+]^	^13^	^43.3^	^15^	^38.5^	^28^	^40.6^
^total^	^30^	^100.0^	^39^	^100.0^	^69^	^100.0^

*YA* young adults, *MA* middle adults, *OA* old adults.

### Social status definition

An essential prerequisite for successful assessment of occupational risk factors impacting the skeletons of Old Kingdom officials who carried out scribal activity is a precise social status definition. The titles held by ancient Egyptians are often indicators of their social status. However, when a title is lacking, the status enjoyed by individuals in society can be identified quite precisely from an archaeological point of view. Based on tomb appearance and architecture, 11 criteria were used for social status assessment of the evaluated individuals: tomb ownership, tomb dimensions, tomb type, chapel dimensions, chapel decoration, the form and execution of false doors, burial shaft depth, the appearance and dimensions of the burial place, the burial manner and the body position (Supplementary Data S3). An overview of the statistical assessment of single criteria decisive for the determination of social status categories is summarized in Supplementary Table S1).

The identities of the high-ranking and higher individuals (their personal names and titles) are known with one exception, while every individual of lower status remains anonymous with no title, and thus evidence of their exact professions is lacking. However, scribal ability was an integral part of the performance of all administrative roles, and not only for those who held the title *sesh* (scribe); the skeletal remains of all individuals of elevated social status have therefore been included in the group of “scribes” for the purposes of this study.

Concerning titles, at least one is attested for 24 (16.6%) of the 69 individuals under study. This set comprises manifold occupations ranging from a vizier, the heads of important administrative departments, priests of the royal mortuary cult and two physicians, to rank-and-file members of institutions or working phyles. A large proportion (10 of 24) worked in the department of legal matters. Six individuals held an actual scribal title: “scribe of treasury” Sekhemka; “scribe of the crews” and “scribe of the archives” Nyankhseshat; “overseer of the scribes of the crew” Nefershepes; “overseer of the scribes of the crew” and “overseer of the royal document scribes” Nefer; “scribe of the royal children” and “scribe of the king” Idu Faaf; and “inspector of the scribes of royal documents in the presence” Inti Pepyankh, who was even buried with scribal tools.

The high-ranking officials were interred in large stone-built mastabas or rock-cut tombs incorporating a decorated funerary chapel equipped with a false door. The deceased, in outstretched position, lay in a spacious burial chamber inside a stone sarcophagus. They were the tomb owners. As a general rule, they headed a crucial administrative department or held an important position in the king’s household.

The individuals categorized as the higher held hierarchically inferior positions to the high-ranking officials. Their burial customs were very similar, but the dimensions of their tombs, chapels and burial chambers could be smaller than those of top-level dignitaries.

The middle-ranking officials might be family members of the tomb owner. They were buried in a roughly dressed sarcophagus, burial pit or a wooden coffin. They usually worked in the area of legal matters and/or as a priest of the royal mortuary cult.

The individuals categorized as being of low-middle are somewhere on the dividing line between the low- and middle-ranking men. Their burials were usually part of that of highly positioned men. The depth of the burial shaft was shallower. They were usually buried in a contracted position in a small burial chamber or in a burial niche. Their identity is known in 4 of 12 cases, and working position in 3 cases (members of the priesthood or working groups providing mortuary cults).

Low-ranking individuals were placed in humble mud-brick tombs with a simple niche instead of a false door. Their body, in a contracted position, was put into a confined niche or simply at the bottom of the shaft. Their identities are unknown. Although the occupation of low-ranking men is not recorded in textual sources, it is very likely that they were involved in the running of their masters’ households, or were members of the priesthood or working groups providing mortuary cults as shown by the depictions on the walls of the tombs^78,79^.

### Distinctive differences between both groups under study

Distinctive differences between the group of scribes and the reference set are: First group (scribes)—the individuals (whose identities are largely known) were mainly buried in large stone-built mastabas or rock-cut tombs, usually placed in a spacious burial chamber in an outstretched position, their bodies protected by a stone sarcophagus or at least a wooden coffin; they were of statuses defined as high-ranking, higher or middle-ranking. Their employment was mainly connected with important administrative offices of the country, which largely involved and required activities related to writing records and creating documents of an administrative nature.

Second group (reference set)—these predominantly anonymous individuals were usually buried in smaller mudbrick tombs or single burial shafts and their bodies of were often put in a contracted position in a confined niche or simply at the bottom of the shaft; they were of statuses defined as low-middle or low. These were usually members of the household or relatives, performing activities related to the running of the household. At the same time, they could also have been members of the priesthood or working groups providing mortuary cults. Thus, scribal activity was not a significant part of their work agenda. From this it can be inferred that low-middle and low status individuals very likely performed different physical activities on a daily basis from officials with a scribal agenda.

### Anthropological methods

Methods used for sex and age-at-death estimation are described in detail in previously published work^77^. The metrical approach^80,81^ was used for primary sex diagnosis where possible, together with morphological evaluation of pelvic structures^82–84^. Where the pelvic bones were missing, the descriptive morphological features^83,84^ and discriminant function analysis of the visually assessed traits^85^ of the skull were used. Metric standards for estimating sex using a discriminant function based on selected measurements of other bones^86–88^ were used as auxiliary methods. The estimation of age-at-death was based on morphoscopic evaluation of morphological changes of pelvic structures: the auricular surface^89,90^, the pubic symphysis^91–93^, and the acetabulum^94^. In addition, changes to the sternal end of the clavicle^95^, the inner architecture of the proximal femur and humerus^96^ and the degree of dental wear^97–99^ were evaluated where possible.

Four groups of changes that might have been related to physical activity were evaluated: entheseal changes, infra-cranial non-metric traits, osteoarthritis of joints of the appendicular skeleton, and degenerative changes of the vertebral column. An overview, including the range of assessed stages and the number of evaluated traits in each category, is presented in Table 4. A total of 1767 items were recorded on each skeleton where preservation allowed. Although some of the evaluated traits show a correlation with age (e.g., entheseal changes, degenerative changes), individuals over 50 years of age were not excluded for the purposes of our study. However, the distribution of individuals in age-at-death categories was statistically tested for scribes and the reference sample.

**Table 4:** Main groups of evaluated skeletal traits including the range of assessed stages and the number of evaluated traits in each category.

Location	Code	Description of trait	Stage	Number of evaluated traits		
				Left	Right	dens/bodies
Appendicular skeleton and skull	EC	Entheseal changes (Supplementary Table S5)	A-B-C	18	18	
	Out	Outer/margin	0–1-2	10	10	
	Inn	Inner/surface	0–1-2	10	10	
	NMT	Non-metric traits (Supplementary Table S6)	0–1	30	30	
		Femur—plaque form	A-B-C	1	1	
		Femur—plaque edge	0–1-2	1	1	
	OA	Osteoarthritis (Supplementary Table S2, Table S3)		72	72	
	MC	Marginal changes	0–1	72	72	
	SC	Surface changes	0–1	72	72	
	NB	New bone	0–1	72	72	
	JC	Joint contour	0–1	72	72	
	Eb	Eburnation	0–1	72	72	
Vertebral apophyseal joints (C1—S1 sup)	OA	Osteoarthritis (Supplementary Table S4)	0–1	49	49	2
	MC	Marginal changes	0–1	49	49	2
	SC	Surface changes	0–1	49	49	2
	Eb	Eburnation	0–1	49	49	2
	SAG	Osteoarthritis; only for cervical	0–1-2–3	14	14	2
Vertebral bodies (C2 inf—S sup)	EC	Entheseal changes (Supplementary Table S4)	0–1-2			46
	SN	Schmorl’s nodes (Supplementary Table S4)	0–1-2			46
	IDD	Intervertebral disc disease (Supplementary Table S4)	0–1			46
	SP	Marginal osteophytes/spondylosis	0–1			46
	SP stage	Stage of spondylosis	1–2-3–4			46
	SC	Surface changes (porosity/piting)	0–1			46
	NB	New bone	0–1			46
	SAG	Osteochondrosis intervertebralis; only for cervical	0–1-2–3			11
				**Total**		**1767**

All joints of the appendicular skeleton (except for hand and foot phalanges), including the temporomandibular joint, were evaluated for OA according to the recommendations of Waldron^14^. A total of 72 articular surfaces on each side were included in the analysis (Supplementary Table S2 and Table S3). Not only the final presence or absence of OA, but also the occurrence of single changes (minor criteria) such as marginal changes (MC), surface changes (SC), new bone (NB), joint contour (JC) and eburnation (Eb) were compared between the two study samples.

Degenerative changes of the vertebral column (C1-S1) were analysed for both apophyseal joints and vertebral bodies (Supplementary Table S4). OA of apophyseal joints were evaluated according to the same method as joints of the appendicular skeleton^14^. Several markers were observed on the vertebral bodies: intervertebral disc disease IDD^14^, inclusive of evaluation of the marginal osteophytes/spondylosis (SP), pitting/porosity and new bone production (SC). Degrees of spondylosis were stated according to Stloukal and Vyhnánek^17^ and were included in the final analysis. Occurrences of Schmorl’s nodes were also recorded^25^. In addition to the methods mentioned above, degenerative changes of the cervical vertebrae were evaluated using a complex method designed only for the cervical spinal section.^13^.

Entheseal changes were recorded on 18 attachment sites of the appendicular skeleton and on 46 vertebral bodies (Supplementary Table S4 and Table S5) according to the method of Villotte^5^, which was current in 2009 when research began. Since most of the skeletons included in this study had already been evaluated at the time of publication of the more recent Coimbra method^100^, and a subsequent study suggest that recording entheseal changes using the revised Coimbra method does not reflect activity more effectively than earlier methods^101^, Villotte’s method was considered appropriate.

NMTs cannot be considered as a homogenous group of markers of uniform origin and/or methodological approach; 28 selected non-metric traits of the infra-cranial skeleton were evaluated and are listed in Supplementary Table S6, including a description of NMT and references to relevant publications based on which evaluated the trait. The presence or absence of NMT was recorded, except for traits at the anterior aspect of the proximal femur (Poirier’s facet, plaque and the cribra/fossa of Allen), where the methodology of Radi et al.^102^ was applied.

### Statistical analysis

Age-at-death distribution in the group of scribes and the reference sample was tested using the Mann–Whitney U test (Python, scipy.stats) and Chi-square test (Python, scipy.stats).

The same tests were used to reveal differences in the incidence of the evaluated changes between scribes and the reference sample. In the case of entheseal changes, all stages (A, B, C respectively 0, 1, 2) were included in the analysis by Mann–Whitney U test, while stages were reduced to presence and absence (0, 1) for testing using the Chi-square test. The Chi-square test was used in the classic form, unless a low sample size or zero frequencies appeared in the contingency table; in such cases, Yates’s correction for continuity was applied. Also, the correction for tied data in the Mann–Whitney U test was applied, since the examined data are categorical.

Considering the discussed issue of using* p* values and null hypothesis significance tests^103^, odds ratios (Python: scipy.stats) and their 95% and 97.5% confidence intervals were calculated to find other ways to interpret the findings.

Since the independence of two variables in a contingency table is equivalent to an odds ratio equal to 1, only the Mann–Whitney *U* test (null hypothesis: the scribes and reference group distribution functions are equal; alternative hypothesis: the scribes and reference group distribution functions are not equal) and the Chi-square test (null hypothesis: the presence of the trait is independent of the scribes and reference group; alternative hypothesis: the presence of the trait depends on the scribes and reference group) are assumed for each trait to establish a family of hypotheses. In other words, both tests are performed for each trait separately, first with their p-values. Then, *the intersection of the two constituent null hypotheses* (the joint null hypothesis) is evaluated against *the union of their constituent alternative hypotheses*, while the family-wise error rate is fixed as the significance level alpha 0.05 in accordance with the Holm-Bonferroni correction. The Holm-Bonferroni correction for *the intersection of two constituent null hypotheses* (as in this case) works as follows: the minimum p-value of their *constituent* p-values is compared to alpha/2 (0.025), and the maximum p-value is compared to alpha (0.05). If at least one of the two constituent null hypotheses is rejected (while the significance level alpha 0.05 for each constituted test is adjusted by applying the Holm-Bonferroni correction), *the union of their constituent alternative hypotheses* is statistically significant, which means that the examined trait is statistically significant for the difference between the scribes and the reference group (where the family-wise error rate did not exceed the predefined significance level alpha). The establishment of the family of hypotheses follows Rubin’s paper^104^ and the desired outcome of the analysis, which is the individual statistical significance of each trait. For these reasons, any other alpha adjustment would be redundant.

Side asymmetry was assessed for all bilaterally occurring traits and was tested using the Sign test and Wilcoxon test (STATISTICA software).

### Supplementary Information

Supplementary Information 1.

Supplementary Information 2.

Supplementary Information 3.

Supplementary Information 4.

## Data Availability

All data and complete results of statistical tests for all traits evaluated are available in the main text or the Supplementary Information. Raw data are available on request from the corresponding author.
