# Supplementary material for: Ancient Egyptian scribes and specific skeletal occupational risk markers (Abusir, Old Kingdom)
Source: Sci Rep. 2024 Jun 27;14:13317. doi: 10.1038/s41598-024-63549-z (PMC11211405; doi:10.1038/s41598-024-63549-z)
Supplement: Supplementary file 1 — Supplementary Information 1. [file 41598_2024_63549_MOESM1_ESM.pdf]

Supplementary Information for

Ancient Egyptian scribes and specific skeletal occupational risk markers (Abusir, Old Kingdom)

Petra Brukner Havelková*, Veronika Dulíková, Šárka Bejdová, Jana Vacková, Petr Velemínský, Miroslav Bárta

*Corresponding author. Email: petra.havelkova@nm.cz

This PDF file includes:

Table S1
Tables S2 to S5
References to Table S2-S5
Table S6
References to Table S6
Data S1 to S3 (legends)

Other Supplementary Information for this manuscript includes the following:

Data S1 to S3

Table S1. Overview of the statistical assessment of single criteria decisive for the determination of social status categories.

status	high-ranking	higher	middle	low–middle	low
tomb owner	5/5	8/9	5/16	3?/12	3?/19
tomb dimensions (m2)	86.58–542.50 / 36.43–51.00	76.50–1,154.00	44.00–390.72	25.08–119.49	3.80–119.49
tomb type	stone mastaba / rock-cut tomb	stone mastaba / rock-cut tomb	stone mastaba / rock-cut tomb / shaft in the court	stone mastaba / rock-cut tomb / mud brick tomb / shaft in the court	mud brick tomb / stone mastaba / shaft in the court
chapel dimension (m2)	6.65–17.55 / 36.43–51.00	1.29–14.33	1.75–14.95	no chapel	no chapel
chapel decoration	5/5	(excellent) low relief / undecorated	no / low relief	no / simple mb niche	no / simple mb niche
false door	5/5	0.00–21.90	no / low relief / sunk relief / with very high-relief statues	no / simple mb niche	no / simple mb niche
depth of the shaft (m)	3.80–11.10	7.70–61.44	4.70–16.50	3.20–7.90	0.00–6.70
burial niche	0/5	0/9	3/16	8; 1 bottom of the shaft	15; 4 bottom of the shaft
burial chamber	5/5	9/9	12/16	3/12	0/19
burial manner	lms sarcophagus, smoothly/roughly dressed	lms sarcophagus, burial pit, wooden coffin	lms sarcophagus, burial pit, wooden coffin, on the floor	bottom of the shaft, wooden coffin, burial pit	bottom of the shaft, wooden coffin , burial pit
position of skeleton	outstretched	outstretched	outstretched	contracted	contracted
dimensions of BC/niche (m3)	8.75–76.70	7.70–53.13	3.70–22.39	0.80–5.14	0.24–4.00
rank titles	*ỉmꜣ-ꜥ, śmr wꜥty, ḥḳꜣt-ꜥ, ḫrp nśty, ḫry-tp nśwt, ỉry ḫt nśwt (ḫr nb.f)*	*ỉry ḫt nśwt*	*śmr wꜥty, ỉry ḫt nśwt, ḫtm(ty)-bỉty*	no	no
other titles	*tꜣyty ṯꜣty (n) zꜣb, ỉmy-rꜣ ḥwt-wrt* 6, *ỉmy-rꜣ kꜣt nbt (nt) nśwt, ỉmy-rꜣ zš(w) ꜥ(w) (nw) nśwt, ỉmy-rꜣ prwy-ḥḏ, ỉmy-rꜣ šnwty, ỉmy-rꜣ zš(w) (n) ꜥprw, ḫrp ꜥḥ, zš mśw-nśwt, ḥḳꜣ ḥwt ꜥꜣt, r Nḫn (n) zꜣb*	*ỉmy-rꜣ zš(w) (n) ꜥprw, ỉmy-rꜣ śḫty-http, ḫtm(w) ḥꜣt ḥtpwt ḏfꜣw bỉty, ḫry śḏm pr-ꜥꜣ, n(y)-nśt-ḫntt, śmśw h(ꜣ)yt (n) zꜣb, r Nḫn (n) zꜣb, śḥḏ zwnw, wr zwnw*	*śmśw h(ꜣ)yt (n) zꜣb, r Nḫn (n) zꜣb, ỉmy-rꜣ wp(w)t ḥtpw, zš pr-ḥḏ, śḥḏ pr-ꜥꜣ, wꜥb nśwt, ḥm-nṯr Mꜣꜥt*	no	unobservable
working activity	vizier, legal matters, organization of royal work, scribe of royal children, priest of royal mortuary cult	legal matters, redistribution, documents, physician, priest of royal mortuary cult	legal matters, privacy of Great House, treasury, priest of royal mortuary cult, priest of divine cult, priest of non-royal cult	organization of labour, phyle-member, priest of royal mortuary cult, priest of divine cult	unobservable

Table S2. Osteoarthritis: overview of evaluated articular surfaces on the upper limbs, including the methods used. All articular surfaces were evaluated separately for the right and left sides. A/P: absence/presence (0/1)

Osteoarthritis of the upper limb

joint	bone	facies	marginal changes		pitting (porosity)		new bone	joint contour	eburnation		final OA
			A/P	stage	A/P	stage	A/P	A/P	A/P	stage	A/P
jaw	scull	mandibular fossa									
	mandible	condylar process									
SCJ	clavicle	sternal facet									
ACJ	clavicle	acromial facet									
	scapula	acromial-clavicular facet									
shoulder	scapula	glenoid fossa									
	humerus	humeral caput									
elbow	humerus	trochlea of humerus									
	humerus	capitulum of humerus									
	radius	caput of radius									
	ulna	trochlear notch									
	ulna	radial notch									
wrist and carpal	radius	lunate art. surf.									
	radius	scaphoid art. surf.									
	radius	ulnar notch									
	ulna	caput of ulna									
	ulna	styloid pr. of ulna									
	lunatum										
	scaphoideum										
	triquetrum										
	pisiforme										
	trapezium										
	trapezoideum										
	capitatum										
	hamatum										
hand	I. metacarpal bone	prox									
		dist									
	II. metacarpal bone	prox									
		dist									
	III. metacarpal bone	prox									
		dist									
	IV. metacarpal bone	prox									
		dist									
	V. metacarpal bone	prox									
		dist									

1) **primary evaluation (Waldron 2009)[1]:**

- eburnation (**Eb**)

or at least two of the following:

- marginal osteophyte/changes (**MC**)
- new bone on the joint surface (**NB**)
- pitting on the joint surface (**SC**)
- alteration in the joint contour (**JC**)

2) **secondary evaluation - stages (Buikstra and Ubelaker 1994)[2]:**

changes	stage	description	affected	
**MC**	1	barely discernible	a	<1/3 of circumference
	2	sharp ridges, sometimes curled with spicules	b	1/3 - 2/3
	3	extensive spicule formation	c	>2/3
	4	ankylosis		
**SC**	1	pinpoint	a	<1/3 of surface
	2	coalesced	b	1/3 - 2/3
	3	both pinpoint and coalesced presented	c	>2/3
**Eb**	1	barely discernible	a	<1/3 of surface
	2	polish only	b	1/3 - 2/3
	3	polish with groove(s)	c	>2/3

Table S3. Osteoarthritis: overview of evaluated articular surfaces on the lower limbs, including the methods used. All articular surfaces were evaluated separately for the right and left sides. A/P: absence/presence (0/1)

Osteoarthritis of the lower limb											
joint	bone	facies	marginal changes		pitting (porosity)		new bone	joint	eburnation		final OA
			A/P	stage	A/P	stage	A/P	A/P	A/P	stage	A/P
hip	pelvis	acetabulum									
	femur	femoral caput									
knee	femur	lateral condyl									
	femur	medial condyl									
	patella	lateral condylar surface									
	patella	medial condylar surface									
	tibia	lateral condyl									
	tibia	medial condyl									
ankle	tibia	medial malleolus									
	tibia	inferior facet									
	fibula	lateral malleolus facet									
	talus	trochlea									
	talus	trochlea medial									
	talus	trochlea lateral									
tarsus	talus	caput									
	talus	ant. mid. articular facet									
	talus	post. articular facet									
	calcaneus	ant. mid. articular facet									
	calcaneus	post. articular facet									
	calcaneus	facies art. cuboidea									
	naviculare	for talus									
	naviculare	for cuneiforme									
	cuneiforme mediale										
	cuneiforme laterale										
	cuboideum	for calcaneus									
	cuboideum	others									
foot	I. metatarsal bone	prox									
		dist									
	II. metatarsal bone	prox									
		dist									
	III. metatarsal bone	prox									
		dist									
	IV. metatarsal bone	prox									
		dist									
	V. metatarsal bone	prox									
		dist									

1) **primary evaluation (Waldron 2009)[1]**

- eburnation (**Eb**)

or at least two of the following:

- marginal osteophyte/changes (**MC)**
- new bone on the joint surface (**NB)**
- pitting on the joint surface (**SC)**
- alteration in the joint contour (**JC)**

2) **secondary evaluation - stages (Buikstra and Ubelaker 1994)[2]**

changes	stage	description	affected	
**MC**	1	barely discernible	a	<1/3 of circumference
	2	sharp ridges, sometimes curled with spicules	b	1/3 - 2/3
	3	extensive spicule formation	c	>2/3
	4	ankylosis		
**SC**	1	pinpoint	a	<1/3 of surface
	2	coalesced	b	1/3 - 2/3
	3	both pinpoint and coalesced presented	c	>2/3
**Eb**	1	barely discernible	a	<1/3 of surface
	2	polish only	b	1/3 - 2/3
	3	polish with groove(s)	c	>2/3

Table S4. Vertebrae: overview of evaluated articular surfaces and changes on the vertebral column, including the methods used. All articular surfaces of apophyseal joints were evaluated separately for the right and left sides. A/P: absence/presence (0/1)

Vertebrae		EC	synovial (apophyseal) joints										intervertebral FC joints (vertebral bodies)						
			left					right					MC		SC	NB	OA	SAGER II.	Schmorl's nodes
			MC	SC	EB	OA	SAG	MC	SC	EB	OA	SAG	A/P	stage					
C1	dens																		
	sup																		
	inf																		
C2	dens																		
	sup																		
	inf																		
C3	sup																		
	inf																		
C4	sup																		
	inf																		
C5	sup																		
	inf																		
C6	sup																		
	inf																		
C7	sup																		
	inf																		
T1	sup																		
	inf																		
T2	sup																		
	inf																		
T3	sup																		
	inf																		
T4	sup																		
	inf																		
T5	sup																		
	inf																		
T6	sup																		
	inf																		
T7	sup																		
	inf																		
T8	sup																		
	inf																		
T9	sup																		
	inf																		
T10	sup																		
	inf																		
T11	sup																		
	inf																		
T12	sup																		
	inf																		
L1	sup																		
	inf																		
L2	sup																		
	inf																		
L3	sup																		
	inf																		
L4	sup																		
	inf																		
L5	sup																		
	inf																		
S1	sup																		

Apophyseal joints/synovial joints (Waldron 2009)[1]

- eburnation (Eb) OR at least two of the following: marginal osteophyte/changes (MC); new bone on the joint surface (NB); pitting on the joint surface (SC); alteration in the joint contour (JC)

Intervertebral joints (vertebral bodies)

- intervertebral disc disease (Waldron 2009)[1]
  - pitting on the inferior or superior surface of vertebral bodies AND marginal osteophyte
- spondylosis (MC stages; Stloukal, Vyhnánek 1976)[3]

1 joint without changes
2 mild changes with osteophytes up to 3mm in size
3 significant changes with osteophytes over 3 mm
4 ankylosis

Schmorl's nodes (Knüsel et al. 1997)[4]

1 less than 2mm deep, covering an area equivalent to less than half of the anteroposterior length of the vertebral body
2 more than 2 mm deep, covering an area of more than half of the anteroposterior length of the vertebral body

Entheseal changes (Villotte 2009)[5]

Cervical vertebrae (Sager 1969)[6]

- apophyseal joints - Articulatio intervertebralis – spondylarthrosis
- osteochondrosis intervertebralis (degeneration of discus)

Table S5. Entheseal changes: overview of evaluated attachment sites, including the methods used. All attachment sites were evaluated separately for the right and left sides.

**Entheseal changes** (Villotte 2009)[5]			LEFT			RIGHT		
code	enthesis	location	Outer	Inner	Stage	Outer	Inner	Stage
HSC	Insertion of the *m. subscapularis*	Humerus: supero-medial part of the lesser tubercle						
HSI	Common insertion of the *mm. supraspinatus and infraspinatus*	Humerus: superior and middle facets of the greater tubercle						
HGP	Insertion of the *m. pectoralis major*	Humerus:intertubercular sulcus of the humerus	/	/		/	/	
HDE	Insertion of the *m. deltoideus*	Humerus: deltoid tuberosity	/	/		/	/	
HEL	Common extensor origin	Humerus: anterior and medial side of the medial epicondyle						
HEM	Common flexor origin	Humerus: anterior and medial side of the medial epicondyle						
RBB	Insertion of the *m. biceps brachii*	Radius: medial half of the tuberosity						
RRP	Insertion of the *m. pronator teres*	Radius: middle of the lateral surface of the body	/	/		/	/	
UTB	Insertion of the *m. triceps brachii*	Ulna: olecranon						
CSB	Common origin of the *m. biceps femoris, semitendinosus* and *semimembranosus*	Os coxae: posterior side of the ischial tuberosity						
FPF	Insertion of the *m. gluteus minimus*	Femur: anterior side of the greater trochanter						
FMF	Insertion of the *m. gluteus medius*	Femur: lateral side of the greater trochanter						
FGF	Insertion of the *m. gluteus maximus*	Femur: gluteal tuberosity	/	/		/	/	
FIP	Insertion of the *m. iliopsoas*	Femur: upper part of the lesser trochanter						
FLA	Insertion of the *m. vastus medialis, m. adductor magnus, m. adductor longus*	Femur: middle third of labium mediale to linea aspera	/	/		/	/	
PQF	*m. quadriceps femoris*	Patella: anterior and upper part of the base						
TSO	Origin of the *m. soleus*	Tibia: soleal line	/	/		/	/	
CTS	Insertion of the *m. triceps surae*	Calcaneus: Achilles tendon attachment (lower part of the dorsal surface)						

References to Table S2 – Table S5:

1. Waldron, T. *Palaeopathology*. (Cambridge University Press, 2009).

2. Buikstra, J. E. & Ubelaker, D. H. *Standards for data collection from human skeletal remains*. (Arkansas Archeological Survey, 1994).

3. Stloukal, M. & Vyhnánek, L. *Slované z velkomoravských Mikulčic*. (Academia, 1976).

4. Knüsel, C. J., Goggel, S. & Lucy, D. Comparative degenerative joint disease of the vertebral column in the medieval monastic cemetery of the Gilbertine priory of St. Andrew, Fishergate, York, England. *Am. J. Phys. Anthropol.* **103**, 481–495. https://doi.org/10.1002/(SICI)1096-8644(199708)103:4<481::AID-AJPA6>3.0.CO;2-Q (1997).

5. Villotte, S. *Enthésopathies et activités des hommes préhistoriques - recherche méthodologique et application aux fossiles européens du Paléolithique supérieur et du Mésolithique.* (Archaeopress, 2009).

6. Sager, P. *Spondylosis cervicalis. A pathological and osteoarchaeological study of osteochondrosis intervertebralis cervicalis, arthrosis uncovertebralis, and spondylarthrosis cervicalis*. (Munksgaard, 1969).

Table S6. List of the evaluated non-metric traits (NMT) including the description of the trait and references to relevant publications.

bone	non-metric trait	description	references
scapula	os acromiale	accessory bone resulting from failure of the acromial apophysis to fuse to the scapula	1-3
pelvis	acetabular crease	linear indentation located in the antero-superior quadrant of the surface of the acetabulum at the level of Byers Feature 17	4
	rounded edged pit	rounded edged pit on the anterosuperior quadrant of the acetabulum	4
	sacro-iliac facet	articular facet on the iliac tuberosity for the sacroiliac ligament	1,2,5
sacrum	sacro-iliac facet	articular facet on the sacral tuberosity for the sacroiliac ligament	1,2,5
proximal femur	Poirier's facet	lateral extension of the anterior portion of the femoral head articular surface toward the anterior aspect of the femoral neck	1,2,6-11
	plaque	imprint located on the anterior margin of the femoral neck, close to the head	1,6,7,10,12
	cribra - anterior cervical imprint	cortical discontinuity in a circumscribed area on the anterior portion of the femoral neck, next to the head	1,6,7,10,12
	Allen's fossa	cortical erosion with exposition of trabecules on the anterior portion of the femoral neck, next to the head	1,6,7,10,12
	posterior cervical imprint	facet on the posterior aspect of the neck, limited laterally by a tubercle bordering the medial margin of the groove for the obturator externus tendon	2,9,13
	exostosis in trochanteric fossa	bilateral exostoses on the superior medial surface of the trochanteric fossa at the insertion of the obturatus externus	1,8,14
distal femur	Charles's facet/prolongation	smooth facet above and behind the medial epicondyle and extending to the adductor tubercle; part of the gastrocnemius bursa	8,9,15
	tibial imprint	depressed, roughened thumb print impression above the posterior aspect of the medial condyle	8,9
	osteochodritic imprint	flattening, depression, plaque or erosion on the posterio-lateral surfaces of the lateral and medial condyles	8,9
	supratrochlear facet/imprint	facet produced by the extension of the superior margin of the lateral trochlear surface to the diaphysis	2,9,13
patella	vastus notch	small notch in the superolateral angle of the patella	1,8,16
	vastus fossa	small depression just anterior to the vastus notch	1
tibia	lateral squatting facet	flexion facet at the anterior surface of the distal end of the tibia at the ankle	15,17-19
talus	forward prolongation of the medial articular surface	various kinds of remodeling occurring on the neck of the talus	17-21
	medial extension of the trochlear surface		
	lateral extension of the trochlear surface		
	medial squatting facet		
	lateral squatting facet		
metatarsal bones	kneeling facet	bony changes on the superior distal surface of the metatarsals	22-24

References to Table S6:

1. Finnegan, M. Non-metric variation of the infracranial skeleton. *J. Anat.* **125**, 23–37 (1978).
2. Kennedy, K. A. R. Skeletal Markers of Occupational Stress in *Reconstruction of Life From the Skeleton* (eds. Iscan, M. Y. & Kennedy, K. A. R.) 129–160 (Alan R. Liss, Inc., 1989).
3. Case, D. T., Burnett, S. E. & Nielsen, T. Os acromiale: Population differences and their etiological significance. *HOMO - Journal of Comparative Human Biology* **57**, 1–18, doi:http://dx.doi.org/10.1016/j.jchb.2005.11.001 (2006).
4. Mafart, B. Description, significance and frequency of the acetabular crease of the hip bone. *Int. J. Osteoarchaeol.* **15**, 208–215, doi:https://doi.org/10.1002/oa.770 (2005).
5. Trotter, M. Accessory sacroiliac articulations in East African skeletons. *Am. J. Phys. Anthropol.* **22**, 137–141, doi:https://doi.org/10.1002/ajpa.1330220213 (1964).
6. Radi, N. *Diversity of the proximal femur in humans: morphological variations of the head-neck junction* Dissertation thesis, Università di Bologna, (2014).
7. Radi, N. *et al.* Variation of the anterior aspect of the femoral head-neck junction in a modern human identified skeletal collection. *Am. J. Phys. Anthropol.* **152**, 261–272, doi:https://doi.org/10.1002/ajpa.22354 (2013).
8. Capasso, L., Kennedy, K. A. R. & Wilczak, C. A. *Atlas of occupational markers on human remains*. (Edigrafital S. P. A., 1998).
9. Kostick, E. L. Facets and Imprints on the Upper and Lower Extremities of Femoral from a Western Nigerian Population. *J. Anat.* **97**, 393–402 (1963).
10. Lawrence, A. B., Sandberg, P. A., Van Gerven, D. P. & Sponheimer, M. Evidence for differences in activity between socioeconomic groups at Kulubnarti, Nubia (550–800 CE), from osseous modifications of the proximal femur. *Int. J. Osteoarchaeol.* **28**, 735–744, doi:https://doi.org/10.1002/oa.2699 (2018).
11. Villotte, S. & Knüsel, C. J. Some remarks about femoroacetabular impingement and osseous non-metric variations of the proximal femur. *Bull. Mém. Soc. Anthropol. Paris* **21**, 95–98 (2009).
12. Angel, J. L. The reaction area of the femoral neck. *Clin. Orthop. Relat. Res.* **32**, 130–142 (1964).
13. Capasso, L. Herculaneum victims of the volcanic eruptions of Vesuvius in 79 AD. *THE LANCET* **356**, 1344–1346, doi:https://doi.org/10.1016/S0140-6736(00)02827-0 (2000).
14. Hawkey, D. E. & Street, S. Activity-induced stress markers in prehistoric human remains from the eastern Aleutian Islands. *Am. J. Phys. Anthropol. Suppl.* **14**, 89, doi:https://doi.org/10.1002/oa.1390050403 (1992).
15. Dlamini, N. & Morris, A. G. An investigation of the frequency of squatting facets in later stone age foragers from South Africa. *Int. J. Osteoarchaeol.* **15**, 371–376, doi:https://doi.org/10.1002/Oa.791 (2005).
16. Messeri, P. Morfologia della rotula nei neolitici della Liguria. *Archivio per l'Antropologia e l'Etnologia* **91**, 1–11 (1961).
17. Boulle, E. L. Evolution of two human skeletal markers of the squatting position: A diachronic study from antiquity to the modern age. *Am. J. Phys. Anthropol.* **115**, 50–56, doi:https://doi.org/10.1002/Ajpa.1055 (2001).
18. Boulle, E. L. Osteological features associated with ankle hyperdorsiflexion. *Int. J. Osteoarchaeol.* **11**, 345–349, doi:https://doi.org/10.1002/oa.572 (2001).
19. Singh, I. Squatting Facets on the Talus and Tibia in Indians. *J. Anat.* **93**, 540–550 (1959).
20. Barnett, C. H. Squatting facets on the European talus. *J. Anat.* **88**, 509–513 (1954).
21. Garg, R., Shekhawat, S., Mogra, K. & Kumar, S. Modifications on Dorsum of Neck of Talus (Squatting Facets and Trochlear Extensions) in Indians. *Acta Med. Int.* **2**, 100–104, doi:https://doi.org/10.5530/ami.2015.1.17 (2015).
22. Ubelaker, D. H. Skeletal evidence for kneeling in prehistoric Ecuador. *Am. J. Phys. Anthropol.* **51**, 679–686, doi:https://doi.org/10.1002/ajpa.1330510417 (1979).

23. Ullinger, J., Kovacik, M. E., Van Gerven, D. P., DeVries, B. & Sheridan, S. G. Metatarsal articular modifications and kneeling in Byzantine monks. *Am. J. Phys. Anthropol. Suppl.* **40**, 1209–1210 (2005).
24. Baker, L. C., Feinman, G. & Nicholas, L. Skeletal evidence for kneeling among prehispanic Zapotec women at the Mitla Fortress in *79th Annual Meeting of the American Association of Physical Anthropology* (Albuquerque, New Mexico, 2010).

Data S1 to S3 (legends):

Data S1. (separate file)

Complete results of the statistical analysis of the evaluated traits on the skull and appendicular skeleton in scribes and the reference group, based on Mann-Whitney (M-W), chi-square ($\chi 2$), confidence interval (CI) and odds ratio (OR) tests and the results of the Mann-Whitney U test of the equality of age distributions between the scribes and the reference group.

EC: entheseal changes; NMT: nonmetric traits; OA: osteoarthritis; MC: marginal changes; SC: surface changes; NB: new bone; JC: joint contour; Eb: eburnation; L: left; R: right; SD: standard deviation; CV: coefficient of variation; U: U-value, test statistic; Z: Z-value, test statistic.

Data S2. (separate file)

Complete results of the statistical analysis of the evaluated traits on the vertebrae in scribes and the reference group, based on Mann-Whitney (M-W), chi-square ($\chi 2$), confidence interval (CI) and odds ratio (OR) tests and the results of the Mann-Whitney U test of the equality of age distributions between the scribes and the reference group.

EC: entheseal changes; OA: osteoarthritis; MC: marginal changes; SC: surface changes; Eb: eburnation; SAG: Sager's method; IDD: intervertebral disk disease; SP: spondylosis; L: left; R: right; SD: standard deviation; CV: coefficient of variation; U: U-value, test statistic; Z: Z-value, test statistic.

Data S3. (separate file)

List of all evaluated individuals, containing 69 adult males, including dating and criteria for social status assessment.
